# TERT and TET2 Genetic Variants Affect Leukocyte Telomere Length and Clinical Outcome in Coronary Artery Disease Patients—A Possible Link to Clonal Hematopoiesis

**DOI:** 10.3390/biomedicines10082027

**Published:** 2022-08-19

**Authors:** Trine B. Opstad, Svein Solheim, Alf-Åge R. Pettersen, Are A. Kalstad, Harald Arnesen, Ingebjørg Seljeflot

**Affiliations:** 1 Center for Clinical Heart Research, Department of Cardiology, Oslo University Hospital Ullevål, Kirkeveien 166, Pb 4950 Nydalen, N-0240 Oslo, Norway; 2 Faculty of Medicine, University of Oslo, 0315 Oslo, Norway

**Keywords:** TERT, TET2, telomere, genetic variation, clonal hematopoiesis

## Abstract

Inherited and acquired mutations in hematopoietic stem cells can cause clonal expansion with increased risk of cardiovascular disease (CVD), a condition known for the clonal hematopoiesis of indeterminate potential (CHIP). Inherited genetic variants in two CHIP-associated genome loci, the telomerase gene telomerase enzyme reverse transcriptase (TERT) (rs7705526) and the epigenetic regulator ten–eleven translocation 2 (TET2) (rs2454206), were investigated in 1001 patients with stable coronary artery disease (CAD) (mean age 62 years, 22% women), with regards to cardiovascular outcome, comorbidities, and leukocyte telomere length. Over 2 years, mutated TERT increased the risk two-fold for major clinical events (MACEs) in all patients (*p* = 0.004), acute myocardial infarction (AMI) in male patients (*p* = 0.011), and stroke in female patients (*p* < 0.001). Mutated TET2 correlated with type 2 diabetes (*p* < 0.001), the metabolic syndrome (*p* = 0.002), as well as fasting glucose, HbA1c, and shorter telomeres (*p* = 0.032, *p* = 0.003, and *p* = 0.016, respectively). In conclusion, our results from stable CAD patients highlight TERTs’ role in CVD, and underline TET2s’ role in the epigenetic regulation of lifestyle-related diseases.

## 1. Introduction

Clonal hematopoiesis (CH) commonly arises due to the age-related acquisition of somatic mutations in hematopoietic stem cells (HSCs) of the bone marrow [1]. The condition increases the risk of hematological malignancies [1], but also cardiovascular disease (CVD) [2]. The phenomenon is known as the clonal hematopoiesis of indeterminate potential (CHIP) [3]. Inherited genetic causes may also lead to CH, by abrogating stem cell function. Single-variant genetic association analyses of CHIP recently identified multiple genome-wide-significant loci with CH association, including the telomerase enzyme reverse transcriptase (TERT) locus at chromosome 5 and the near ten-eleven translocation 2 (TET2) locus at chromosome 4. Germline, i.e., inherited genetic variants in both TERT and TET2 genes have been associated with an increasing risk of CHIP development and malignant hematopoiesis, respectively [4,5].

The telomerase enzyme, known to be active in germ cells, pluripotent embryonic stem cells and hematopoietic progenitor cells, seems also to be active in differentiated, non-dividing, somatic cells in the cardiovascular system, sustaining the role of telomere biology in CVD [6]. Telomeres protect the ends of chromosomes, consisting of repeated DNA sequences (TTAGGG) and associated proteins [7]. Telomerase synthesizes telomeric DNA to compensate for and counteract the loss of sequences upon cell division; however, its overexpression may lead to cancer [8]. As telomere shortening in general is related to organismal and cardiovascular ageing, a delicate balance in telomere stability may thus be required to achieve healthy ageing. We recently reported shorter leukocyte telomere length (LTL) in young and elderly healthy subjects with hereditary coronary heart disease [9]. Short telomeres in blood cells may lead to somatic mutations, and potentially CH, and vice versa, manifested CHIP may shorten LTL [10]. Furthermore, genetically predicted longer telomere lengths were associated with altered clonal somatic copy number in peripheral leukocytes [5], potentially also predisposing CH; thus, a bidirectional causality between telomere lengths and CHIP has been suggested [10]. Several TERT germline mutations predisposed to CH have been identified, including the single-point rs7705526 mutation, previously associated with increased leukocyte telomere length (LTL), myeloproliferative neoplasms, and increased risk of developing CHIP [4,11,12].

TET2 is expressed in hematopoietic cells and is important for normal hematopoiesis. The enzyme regulates gene expression via the DNA demethylation of cytosine on DNA targets, and oxidizes 5-methyl cytosine to 5-hydroxymethylcytosine, thus epigenetically modulating the genome [13]. The TET family of proteins are thought to be involved in the pathogenesis of complex metabolic-related diseases [14] and TET2 is involved in telomere maintenance and chromosomal stability. Most TET2 somatic and germline mutations are *loss-of-function* mutations, located in the enzymes’ catalytic domain in the C-terminal part, consequently leading to hyper-methylated DNA [13]. Somatic mutations inducing TET2 deficiencies may, in addition to malignancies, also cause atherosclerosis and adverse CVD outcomes, mediated by macrophage pro-inflammatory activation and interleukin upregulation [15,16]. Several germline TET2 mutations have been investigated according to myelodysplastic malignancies, including the Ile1762Val variant in exon 11 [13,17]. So far, no germline TET2 mutation has been reported with regards to CVD.

We aimed to investigate two genetic variants, reported as germline mutations [4,5], that could potentially induce somatic mutations in HSCs ensuing CHIP development: the TERT intron 5 rs7705526 variant and the TET2 p.Ile1762Val rs2454206 missense mutation in patients with stable coronary artery disease (CAD). Our hypothesis was that these variants could affect LTLs, cardiometabolic status, and clinical outcome, with potential causality related to CHIP or its development.

## 2. Materials and Methods

### 2.1. Study Population

This study is a post-hoc analysis of 1001 angiographically verified CAD patients enrolled in the Aspirin Non-Responsiveness and Clopidogrel Endpoint Trial (ASCET), with a mean age of 62 years (22% were females and 97% were of western European descent) [18]. Patients were followed for a minimum of 2 years, and the primary clinical endpoint major adverse events (MACEs) included the first event of either a nonfatal acute myocardial infarction (AMI), unstable angina pectoris (UAP), stroke, and all-cause mortality. An endpoint committee evaluated the endpoints without access to laboratory data. No patients were lost to follow-up, and if they were unable to attend the final visit, clinical endpoints were recorded on request.

At inclusion, clinical subgroups were defined as follows: previous MI, as recorded by patients medical files; hypertension (HT), i.e., individuals previously diagnosed or treated HT; diabetes, i.e., individuals with treated type 2 diabetes and/or fasting glucose > 7.0 mmol/L; and metabolic syndrome (Mets), according to modified NCEP ATP III criteria [19]. These was a threshold value of at least three of the following: (1) waist circumferences ≥ 102 cm in men and ≥88 cm in women; (2) triglycerides ≥ 1.69 mmol/L; (3) HDL-cholesterol < 1.04 mmol/L in males and <1.29 mmol/L in females; (4) blood pressure ≥ 130/85 mm Hg or diagnosed or treated HT; and (5) fasting glucose ≥ 6.1 mmol/L.

The Regional Committee of Medical Research Ethics South-Eastern Norway approved the ASCET study. All research in the present study was performed in accordance with relevant guidelines and regulations. The participants conformed to the Declaration of Helsinki and written informed consent was obtained from all patients. The ASCET study was registered at clinical trial.gov, http://www.clinicaltrial.com (accessed on 22 September 2005), with the identification number NCT00222261.

### 2.2. Laboratory Methods

Blood samples were collected at baseline between 8.00 and 10.00 a.m. in fasting conditions. Serum was prepared by centrifugation within 1 h at 2500× *g* in 10 min for routine analyses. EDTA whole-blood samples were kept frozen at −80 °C until DNA extraction, performed with the MagNA Pure LC DNA Isolation Kit on the MagNA Pure LC Instrument (Roche Diagnostics, GmbH, Mannheim, Germany). Extracted DNA was tested for purity and quantity using a NanoDrop 1000 spectrophotometer (Thermo Scientific, Wilmington, DE, USA) and stored at −80 °C. LTL analysis was performed in a subgroup of the first included patients (*n* = 262).

### 2.3. Genotype Analysis

The allelic discrimination of TERT C/A (rs7705526) and TET2 p.Ile1762Val A/G (rs2454206) variants was performed with real-time PCR on the ViiA7 instrument (Applied Biosystems, Thermo Fisher Scientific), using the TaqMan single-nucleotide polymorphism (SNP) assays ID C_189441058_10 and ID C_11566753_20, respectively (Life Technologies dba Invitrogen, Pleasanton, CA, USA). The TaqPath ProAmp Master Mix was used for the TERT assay and TaqMan Universal PCR Master Mix for the TET2 assay. Non-template samples were included in each run to exclude contamination of samples, and 5% of the samples were re-ran as quality control, with 99% concordance.

### 2.4. Leukocyte Telomere Length (LTL) Determination

An equal amount of extracted DNA per experiment (2 ng/μL) was used to measure LTLs using a single-plex quantitative RT- PCR [20]. PCR amplification was performed on the ViiA7 instrument (Applied Biosystems by Life Technologies, Foster City, CA, USA), using telomere-specific primers (Invitrogen by Thermo Fisher Scientific, Waltham, MA, USA) (Supplementary Table S1) and GoTaq PCR Master Mix (Promega, Madison, WI, USA). LTLs were relatively quantified (RQ) to the single-copy gene (SCG) SB34 (Invitrogen by Thermo Fisher Scientific) with specific SCG primers (Supplementary Table S1) and an internal reference sample. The primers for both targets were diluted to a final concentration of 4 pmol/µL. PCR conditions for both targets were as follows: an initial step at 95 °C for 10 min followed by 40 cycles of 95 °C for 15 s and 60 °C for 1 min. A template negative control was included in each run. Each sample was run in triplicates and individual amplification curves for all samples of both assays were carefully validated. Technical triplicates with a SD exceeding 0.5 Ct were excluded from the analysis, with two remaining valuable parallels.

### 2.5. Statistical Analysis

Data were presented as mean (+/−SD), median (25th, 75th percentile), or proportions (%), as appropriate. The unpaired two-sampled Student *t*-test and one-way ANOVA were used for the analysis of normal distributed continuous data, and the Mann–Whitney U test and Kruskal–Wallis test were used for skewed continuous data, when appropriate, for comparisons between two or more groups, respectively. Chi-squared tests were used to compare the proportions. A binary logistic regression model was used in association between the TERT variant and clinical endpoints, adjusting for age and sex (included in the model by convention), and previous MI and stroke (more frequent present at inclusion in subjects suffering from new clinical events). The TERT and TET2 variants were tested with the Hardy–Weinberg equilibrium (x^2^ > 0.05, both). The level of statistical significance was set to *p* < 0.05. SPSS version 26 (SPSS Inc., Chicago, IL, USA) was used for all statistical analyses.

## 3. Results

In the investigated population, 106 MACEs were recorded after 2 years (AMI (*n* = 36), stroke (*n* = 28), UAP (*n* = 33) and deaths (*n* = 9)). Baseline characteristics according to the presence of endpoints or not are presented in Table 1, with previous MI and stroke being more frequent in patients suffering from a new clinical event. Independently of MACEs, the mean age in the population was 62 years, and 22% were women. The presence of comorbidities was as follows: 20% had type 2 diabetes, 24% had Mets, 56% had hypertension, and 20% were current smokers. 98% of patients were on statin treatment, and many used anti-hypertensive medications.

### 3.1. Frequencies of the TERT and TET2 Mutations

DNA was available in 995 samples and the investigated variants were successfully analyzed in all, except for one TERT measurement. Genotypes and variant allele frequencies (VAFs) in the CAD subjects, stratified by sex, are presented in Table 2. VAFs for the TERT C/A and TET2 p.Ile1762Val A/G variants were 0.324 and 0.346, respectively. A higher frequency of the TERT variant was observed in men vs. women (*p* = 0.033).

### 3.2. Presence of the TERT and TET2 Mutations as Related to Clinical Outcome and Comorbidity

**Figure 1:**
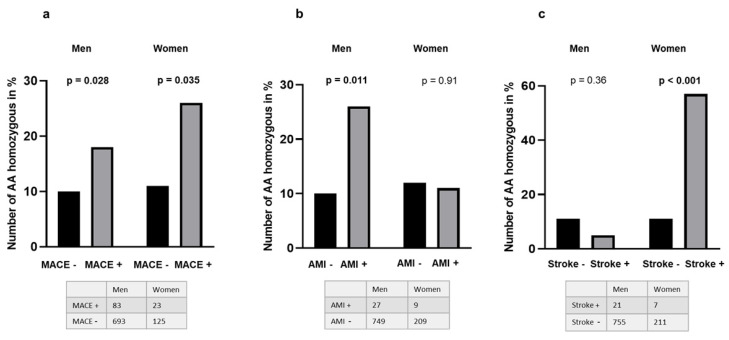
Association of the TERT rs7705526 mutation with MACE in certain subcategories. Tables underneath each figure denote actual numbers in different groups. (**a**) Frequency of TERT rs7705526 (C/A) homozygous subjects (AA) according to major clinical events (MACEs), separated by gender. Black and gray columns represent the % of AA homozygous without and with MACE, respectively. (**b**) Frequency of TERT rs7705526 (C/A) homozygous subjects (AA) according to acute myocardial infarction (AMI), separated by gender. Black columns represent the % of AA homozygous without AMI in both gender. The gray columns represent the % of AA homozygous suffering from AMI in men and women, respectively. (**c**) Frequency of TERT rs7705526 (C/A) homozygous subjects (AA) according to stroke, separated by gender. Black columns represent the % of AA homozygous patients without stroke. The gray columns represent the % of AA homozygous suffering from strokes in women and men, respectively.

The variants’ influence on clinical outcome is presented in Table 3. An increasing number of TERT variant alleles (A) correlated with an increased risk of MACEs (*p* = 0.010). In terms of categorization, the AA genotype vs. the CC and CA genotypes increased the risk further (*p* = 0.003), with an OR of 2.2 (95 % confidence interval [CI] of 1.3, 3.7), which was still significant when adjusting for age, sex, previous MI, and stroke (OR = 2.2, 95% CI [1.3, 3.7], *p* = 0.004). As the TERT mutation was more frequent in men, we analyzed the data separately. The results are illustrated in Figure 1, showing an increased risk of MACE in both genders: in men with an OR = 2.0 (95% CI (1.1, 3.6), *p* = 0.028) and in women with an OR = 2.9 (95% CI (1.0, 8.), *p* = 0.035) (Figure 1a). With the mutation, an increased risk of new-onset AMI was found in men only (OR = 3.0, 95% CI [1.2, 7.3], *p* = 0.011, *n* = 27) (Figure 1b), whereas an increased risk of new onset stroke was observed in women only (OR = 10.9, 95% CI [2.3, 51.8], *p* < 0.001, *n* = 7) (Figure 1c). The TERT mutation was not associated with previous MI and stroke, or other comorbidities (Table 3).

The TET2 mutation was not associated with MACE (Table 3), but was significantly and more frequently present in patients with type 2 diabetes (*p* < 0.001) and with Mets (*p* = 0.002). In line with this, significant associations were observed with increasing fasting glucose levels across AA-AG-GG genotypes: mean (SD) 6.0 (0.1)-6.0 (1.0)-6.5 (0.2) mmol/L (*p* = 0.032) and increasing HbA1c levels 5.9 (0.04)-6.0 (0.04)-6.2 (0.11)% (*p* = 0.003).

### 3.3. TERT and TET2 Mutations as Related to Leukocyte Telomere Lengths (LTLs)

**Figure 2:**
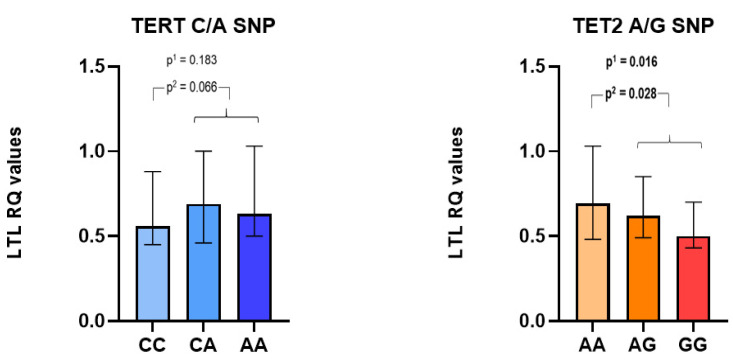
Relatively quantified (RQ) leukocyte telomere length (LTL), related to TERT rs7705526 (C/A) and TET2 rs2454206 (A/G) genotypes. p^1^ values refer to the difference in LTLs between genotypes, whereas p^2^ values refer to differences in LTLs between the presence of the variant allele compared to the wild type. In this subset, LTLs were not associated with MACE (*n* = 34), independent of TERT and TET2 genotypes (median RQ level (25, 75 percentiles): 0.67 (0.51, 0.91) as compared to without MACE (*n* = 228): 0.61 (0.46, 0.96)).

In Figure 2, we illustrated the relationship between TERT and TET2 mutations, respectively, and LTLs (RQ levels) measured in the subset of CAD patients (*n* = 262). The TERT A allele correlated with borderline significant longer LTLs (*p* = 0.066), as compared to the CC wild type, with an overall non-significant difference in LTLs between genotypes (*p* = 0.183). LTLs were significantly shorter with an increasing number of TET2 variant G-alleles (*p* = 0.016).

## 4. Discussion

The main finding in our study is that the selected germline intronic TERT C/A variant (rs7705526) correlated with composite clinical endpoints during two-year follow-up in patients with stable CAD, with an increased risk of AMI exclusively in men and an increased risk of stroke exclusively in women. The investigated TET2 genetic variant (rs2454206), with an amino acid substitution of isoleucine to valine at position 1762, correlated with shorter telomeres and the presence of type 2 diabetes and Mets, and accordingly with fasting glucose and HbA1c levels.

By simultaneously analyzing germline and somatic mutations with blood-derived whole-genome sequencing (WGS), recent comprehensive studies have demonstrated that germline mutations influence the acquisition of somatic mutations in blood cells [11,12,21]. Inherited causes of CHIP include multiple genetic variants at the TERT and TET2 locus [4]. The investigated TERT genetic variant (rs7705526) in intron 5 is in strong linkage disequilibrium (r^2^ = 0.55) with the TERT genetic variant rs34002450 in intron 3, both identified as lead variants in the TERT locus, and as germline genetic determinants of CHIP [4,21], the latter with a 1.3-fold increased risk of CHIP development (TOPMed project) [4]. The rs34002450 presented with a 1.37-fold increased risk of developing CH in the Iceland deCODE genetics project [21]. Thus, the TERT-CHIP association may indicate the role of telomerase activity in CH. We observed borderline significantly longer LTLs in TERT rs7705526 A-allele carriers, as previously reported by others [5,11]. We also observed that LTLs, independently of the investigate gene variants, were not linked to MACE, and were measured in a subpopulation, indicating underpowered analysis and/or the fact that the already-manifested CAD status in our population may have affected the results. Telomerase overexpression has been reported to transform cultured cells into cancerous cells [22], and failure of the TERT gene may deteriorate genome integrity, enabling the acquisition of somatic mutations in hematopoietic cells and the further development of CHIP. As TERT is also active in the cells of the cardiovascular system, any interference with TERT activity may contribute to CVDs [6]. The TERT rs7705526 variant has been shown to lead to the CHIP-related somatic mutation JAK2 p.V617F [23], supporting the possibility of the causational TERT A-allele-CHIP development in our CAD population.

Although numbers are low, the observed sex dimorphism in the frequency of the TERT rs77055526 variant and in the type of MACE may indicate different underlying pathophysiological mechanisms in men and women with regards to the involvement of TERT in CVD development, in need of further investigation. Indeed, the use of hormone replacement therapy or merely a postmenopausal state might have influenced the association found between the onset of strokes in women and the TERT mutation. Unfortunately, we do not have data to explore any influence.

TET2 is one of the most common somatically mutated genes in CH and CHIP [1,21,24] and multiple germline TET2 mutations have been investigated according to myeloid malignancies [13,17,25]. The TET2 p.Ile1762Val genetic variant is located in the catalytic domain of the TET2 gene; thus, an eventual loss of TET2 function may lead to DNA hypermethylation and subsequent altered gene expression in blood cells. We observed that the variant was present at a significantly higher frequencies in both type 2 diabetes and Mets subjects, with altered glucose and HbA1c levels. Inherited TET2 mutations ensuing life-long epigenetic changes may have altered pathways in glucose regulation. TET2 has been suggested to facilitate the transcriptional activity of peroxisome proliferator-activated receptor gamma (PPARƴ), involved in insulin sensitivity; thus, a TET2 *loss-of-function* may have promoted insulin resistance [26]. The TET2 p.Ile1762Val variant has also previously been reported to correlate with liver PPARƴ coactivator 1 alpha (PGC1A)-methylation levels and non-alcoholic fatty liver disease [27]. TET2 has also been proposed to regulate PPARƴ transcription in adipocytes [26], which can partly explain the observed Mets and type 2 diabetes association in the present study. The TET2 rs2454206 genetic variant has also been reported to correlate with diabetes [14], and mice experiments suggest that TET2 *loss-of-function*-driven clonal hematopoiesis can contribute to insulin resistance and type 2 diabetes [28].

Both the TERT mutation (rs7705526) and the TET2 (rs2454206) are reported to significantly correlate with levels of blood pressure in the Common Metabolic Diseases Knowledge Portal (hugeamp.org). The lack of these associations in our study might be due to medication status and/or the population itself consisting exclusively of patients with stable CAD.

The TET2 variant correlated with shorter telomeres, to our knowledge not previously reported in humans. As TET enzymes are important for telomere stability, the investigated TET2 variant may potentially have induced TET2 deficiency and subsequently telomere loss [29]. Experiments with TET2-depleted mice resulted in shorter telomeres, explained by an upregulation of DNA methyltransferase, which decreases 5-hydroxymetylcytosine levels and increases the methylation status at sub-telomeres, the region between telomeric caps and chromatin [29]. Aberrant methylation on sub-telomeric DNA may have certain effects on telomere lengths, correlating with age-related diseases [30], underpinning the connection between diabetes and Mets. The detected shorter telomeres with the TET2 variant in our population may have hypothetically induced somatic mutations, CH and CHIP-related CAD, which indirectly may have accelerated telomere attrition, although any causality cannot be drawn.

Our study has several limitations. We investigated candidate SNPs with only two variants; thus, any influence of other known TERT and TET2 genetic variants is not explored. The population consisted exclusively of medically treated patients with stable CAD. With the low numbers of LTL analyses, these results should be regarded as explorative and hypothesis-generating, and must be taken with caution. As others have reported an association between the TERT rs7705526 mutation and longer telomeres, our lack of a statistical significant association ensues the possibility of a type 1 statistical error. Its effect on cardiovascular outcome may however underpin the idea that longer telomeres may induce CHIP development, and herein the observed increased risk of MACE, despite other mechanisms of the observed associations, cannot be excluded. The low number in studied subgroups may have also influenced results with regards to clinical endpoints.

## 5. Conclusions

The TERT rs7705526 mutation correlated with an increased risk of clinical adverse events in our CAD population, potentially related to CHIP development. The TET2 p.Ile1762Val missense mutation rs2454206 correlated with type 2 diabetes and Mets, along with dysregulated glucose metabolism, illustrating epigenetic regulation as a bridge between inherited and environmental causes in the development of disease. The associated shorter telomeres may reflect their manifested CAD.

## Figures and Tables

**Table 1:** Baseline characteristics according to presence of clinical composite endpoints after 2 year in the CAD population.

	With Endpoints (*n* = 106)	Without Endpoint (*n* = 895)	*p*
Age (years, mean (range))	63 (41–80)	62 (36–81)	0.499
Men/women *n* (%)	83/23 (78/22)	700/195 (78/22)	0.983
Type 2 diabetes Mellitus *n* (%)	24 (23)	176 (20)	0.469
Previous myocardial infarction *n* (%)	57 (54)	380 (43)	**0.026**
Metabolic syndrome (%)	25 (24)	219 (25)	0.836
Previous stroke *n* (%)	6 (6)	21 (2.3)	**0.047**
Hypertension *n* (%)	63 (59)	493 (55)	0.394
SBP mm/Hg	140 (125, 150)	140 (125, 150)	0.831
DBP mm/Hg	80 (75, 90)	80 (75, 90)	0.616
Current smokers *n* (%)	23 (22)	180 (20)	0.666
BMI (kg/m^2^) ^a^	27.4 (4.0)	27.7 (9.4)	0.742
Total cholesterol (mmol/L)	4.5 (1.0)	4.6 (1.0)	0.877
HDL cholesterol (mmol/L)	1.3 (0.4)	1.3 (0.4)	0.898
LDL cholesterol (mmol/L)	2.5 (0.8)	2.5 (0.8)	0.758
Triglycerides (mmol/L) ^a^	1.5 (0.9)	1.6 (1.1)	0.887
Fasting glucose (mmol/L)	6.1 (1.7)	6.0 (1.9)	0.914
HbA1c (%)	6.05 (0.87)	5.97 (0.91)	0.42
Medication (%)			
Statins	98	99	0.524
β-lockers	74	76	0.867
Nitrates	27	21	0.145
ACE inhibitors	31	26	0.32
ARB	26	24	0.711
CCB	27	25	0.656
Diuretics	26	22	0.417

Values are mean (SD) or numbers (%) if not otherwise stated, ^a^ median levels (25, 75. percentile). SD: standard deviation, SBP: systolic blood pressure, DBP: diastolic blood pressure, BMI: body mass index, HDL: high-density lipoprotein, LDL: low-density lipoprotein, ACE: angiotensin-converting enzyme, ARB: angiotensin receptor blocker, CCB: calcium channel blocker. *p*-values are chi-square test for categorical variables and *t*-test or Mann–Whitney test for continuous variables, referring to differences between patients with and without clinical endpoint. Bold text represents significant *p*-values (*p* < 0.05).

**Table 2:** Frequencies of the TERT rs7705526 and TET2 rs2454206 genetic variants in CAD patients, stratified by gender.

	TERT Genotypes					TET2 p.Ile1762Val				
	CC	CA	AA	VAF	*p*	AA	AG	GG	VAF	*p*
CAD patients	461	421	112	0.324		434	434	127	0.346	
Men (*n* = 777)	346	345	85	0.333	**0.033**	342	339	96	0.342	0.74
Women (*n* = 218)	115	76	27	0.299		92	95	31	0.360	

VAF; variant allele frequency *p*-values refer to difference in VAF between sex. Bold text represents a significant *p*-value (*p* < 0.05).

**Table 3:** The presence of the TERT rs7705526 and TET2 rs2454206 genetic variants in the CAD population, as related to clinical outcome after 2 years, and comorbidity at baseline.

Clinical Status	*n* ^a^		TERT Genotypes					TET2 Genotypes				
			CC	CA	AA	VAF	*p*	AA	AG	GG	VAF	*p*
Composite endpoint	Yes	106	41	44	21	0.406	**0.010**	49	46	11	0.321	0.70
	No	889	420	377	91	0.315		385	388	116	0.348	
Diabetes type 2	Yes	198	93	85	20	0.315	0.85	198	74	82	0.417	**<0.001**
	No	797	368	336	92	0.327		360	352	85	0.328	
Metabolic syndrome	Yes	242	114	105	23	0.312	0.60	97	99	47	0.397	**0.002**
	No	751	347	315	89	0.329		337	334	80	0.330	
Previous MI	Yes	433	209	173	51	0.318	0.40	190	193	51	0.341	0.69
	No	561	252	248	61	0.330		244	241	76	0.350	
Previous Stroke	Yes	26	16	7	3	0.250	0.25	8	12	6	0.462	0.196
	No	967	445	413	109	0.327		425	422	121	0.343	
Hypertension	Yes	554	242	245	67	0.342	0.153	226	249	80	0.369	0.063
	No	440	219	176	45	0.302		208	185	47	0.317	

^a^ actual numbers in different subgroups. VAF; variant allele frequency, MI; myocardial infarction, *p*-values refer to difference in genotype frequencies between actual subgroups, using the chi-square test. Bold text represents significant *p*-values (*p* < 0.05).

## Data Availability

The data presented in this study are available on request from the corresponding author. The data are not publicly available due to the privacy of the patients.

## Supplementary Materials

The following supporting information can be downloaded at: https://www.mdpi.com/article/10.3390/biomedicines10082027/s1. Supplementary Table S1: Nucleotide sequence for the telomere and single-copy gene analyses.
