# Supplementary material for: TERT and TET2 Genetic Variants Affect Leukocyte Telomere Length and Clinical Outcome in Coronary Artery Disease Patients—A Possible Link to Clonal Hematopoiesis
Source: Biomedicines. 2022 Aug 19;10(8):2027. doi: 10.3390/biomedicines10082027 (PMC9406025; doi:10.3390/biomedicines10082027)
Supplement: Supplementary file 1 [file biomedicines-10-02027-s001.zip › biomedicines-1827075-supplementary-updated.pdf]

Supplementary Material

Table S1. Nucleotide sequence for the telomere and single copy gene analyses.

PCR primers	Oligomer sequences (5’ to 3’)
Telomere fw.	CGG TTT GTT TGG GTT TGG GTT TGG GTT TGG GTT TGG GTT
Telomere rev.	GGC TTG CCT TAC CCT TAC CCT TAC CCT TAC CCT TAC CCT
Single copy gene (36B4) fw	CAG CAA GTG GGA AGG TGT AAT CC
Single copy gene (36B4) rev	CCC ATT CTA TCA TCA ACG GGT ACA A

fw; forward, rev; reverse
